# Systemic lupus erythematosus: pathogenesis and targeted therapy

**DOI:** 10.1186/s43556-024-00217-8

**Published:** 2024-10-30

**Authors:** Xu Su, Hui Yu, Qingqiang Lei, Xuerui Chen, Yanli Tong, Zhongyang Zhang, Wenyong Yang, Yuanbiao Guo, Liangbin Lin

**Affiliations:** 1 Medical Research Center, College of Medicine, The Third People’s Hospital of Chengdu (Affiliated Hospital of Southwest Jiaotong University, Southwest Jiaotong University, Chengdu, 610031 Sichuan China; 2 Department of Urology, The Third People’s Hospital of Chengdu, The Affiliated Hospital of Southwest Jiaotong University, Chengdu, 610014 China; 3 Center of Bone Metabolism and Repair, Department of Wound Repair and Rehabilitation Medicine, State Key Laboratory of Trauma, Burns and Combined Injury, Trauma Center, Research Institute of Surgery, Daping Hospital, Army Medical University, Chongqing, 400000 China; 4 Université Paris Cité, INSERM U1151, CNRS UMR8253, Institut Necker Enfants Malades, Paris, F-75015 France; 5 Department of Health Technology, The Danish National Research Foundation and Villum Foundation’s Center IDUN, Technical University of Denmark, Kgs. Lyngby, Denmark; 6 Department of Neurosurgery, The Third People’s Hospital of Chengdu, The Affiliated Hospital of Southwest Jiaotong University, Chengdu, 610014 China; 7 Obesity and Metabolism Medicine-Engineering Integration Laboratory, Department of General Surgery, The Third People’s Hospital of Chengdu, The Affiliated Hospital of Southwest Jiaotong University, Chengdu, 610031 China; 8 The Center of Gastrointestinal and Minimally Invasive Surgery, Department of General Surgery, The Third People’s Hospital of Chengdu, The Affiliated Hospital of Southwest Jiaotong University, Chengdu, 610031 China

**Keywords:** Systemic lupus erythematosus, Autoimmune diseases, Pathogenesis, Targeted therapeutic strategies

## Abstract

Systemic lupus erythematosus (SLE) is a multifaceted autoimmune disorder characterized by dysregulated immune responses and autoantibody production, which affects multiple organs and varies in clinical presentation and disease severity. The development of SLE is intricate, encompassing dysregulation within the immune system, a collapse of immunological tolerance, genetic susceptibilities to the disease, and a variety of environmental factors that can act as triggers. This review provides a comprehensive discussion of the pathogenesis and treatment strategies of SLE and focuses on the progress and status of traditional and emerging treatment strategies for SLE. Traditional treatment strategies for SLE have mainly employed non-specific approaches, including cytotoxic and immunosuppressive drugs, antimalarials, glucocorticoids, and NSAIDs. These strategies are effective in mitigating the effects of the disease, but they are not a complete cure and are often accompanied by adverse reactions. Emerging targeted therapeutic drugs, on the other hand, aim to control and treat SLE by targeting B and T cells, inhibiting their activation and function, as well as the abnormal activation of the immune system. A deeper understanding of the pathogenesis of SLE and the exploration of new targeted treatment strategies are essential to advance the treatment of this complex autoimmune disease.

## Introduction

Systemic lupus erythematosus (SLE) is a quintessential autoimmune disease, marked by recurring episodes and periods of remission, with the potential to cause extensive damage to multiple organ systems and tissues. This condition primarily affects the kidneys, central nervous system, joints, and skin [1]. A defining characteristic of SLE is the production of autoantibodies that target self-antigens, leading to the creation of immune complexes. These complexes accumulate within blood vessels, triggering intense inflammatory reactions that can lead to the dysfunction of various organ systems. The multifaceted nature of SLE and its propensity for diverse organ involvement pose significant therapeutic challenges, necessitating a comprehensive understanding of the underlying immunopathogenic mechanisms to develop targeted and effective treatment strategies [2, 3].

Recent medical advances have shed light on the etiology of SLE, with increasing recognition of dysregulated immune mechanisms involving both the adaptive and innate immune compartments. While the current management of SLE predominantly relies on broad-spectrum immunomodulatory and immunosuppressive agents [4], the therapeutic landscape is evolving with the development of novel therapeutics tailored toward precise immunologic epitopes, some of which have garnered endorsement from regulatory authorities [5, 6].

In this review, we provide a comprehensive overview of the pathogenic mechanisms and contemporary treatment modalities for SLE, encompassing both traditional and emerging therapeutic strategies. The onset of SLE is intricately linked to the interplay between immune dysregulation, genetic predisposition, the collapse of immune tolerance, and a series of environmental triggers. These complex interactions ultimately result in the production of pathogenic autoantibodies. These lupus-associated autoantibodies collaborate with adaptive and innate immune responses, facilitating the accumulation of immune complexes in various tissues and organs. This process triggers acute and chronic inflammation, ultimately leading to damage to the affected organs. Furthermore, we conduct an in-depth assessment of the therapeutic efficacy of conventional approaches and newly emerging targeted therapies for SLE. We also offer insightful projections on the prospective evolution of innovative treatment options in the field. SLE, as a systemic autoimmune disease with complex pathophysiological characteristics, poses significant challenges to current therapeutic strategies. Accordingly, gaining an in-depth comprehension of SLE’s pathogenic mechanisms and advancing precise, potent targeted treatments are of utmost importance in clinical practice.

## Pathogenesis of SLE

**Fig. 1:**
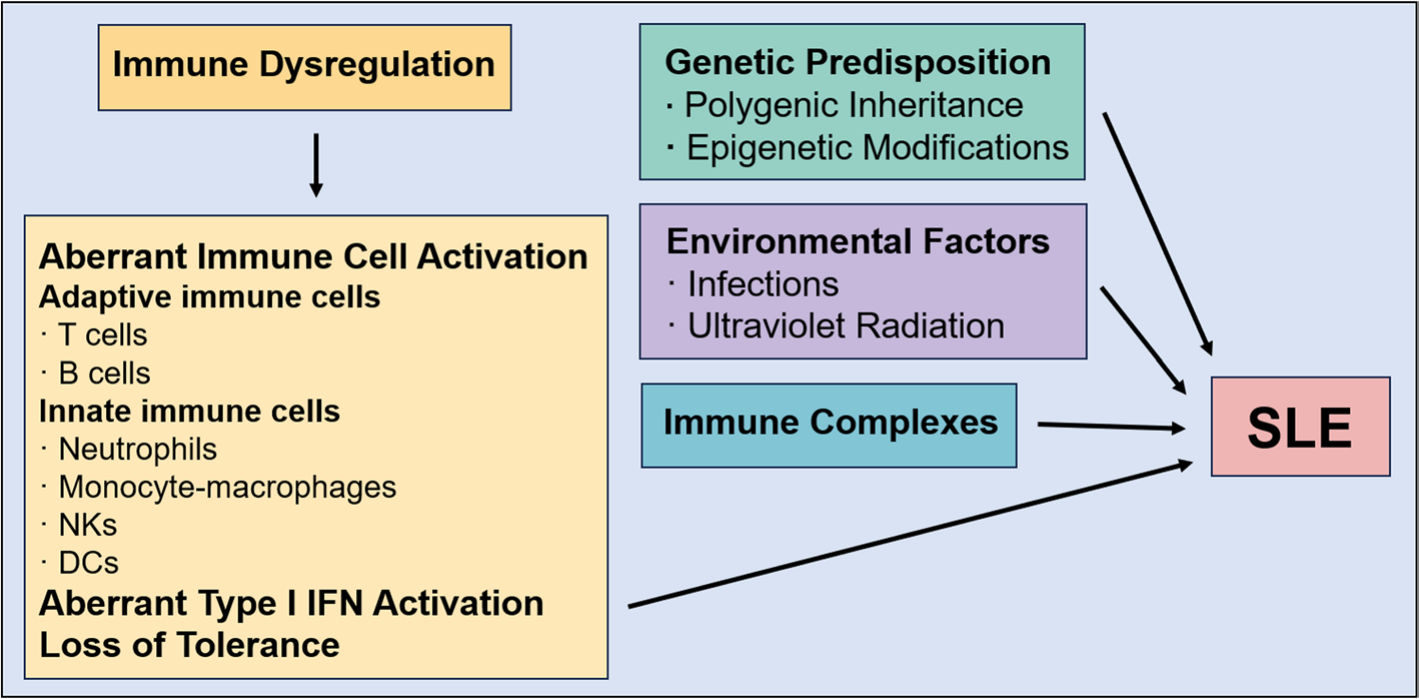
The pathogenesis of SLE. The pathogenesis of SLE is complex and involves immune dysregulation, genetic predisposition, environmental factors and immune complexes and tissue injury

SLE is characterized by its chronic nature and the multifaceted impact it has on various organs. The onset of SLE is linked to the collapse of immunological tolerance coupled with the interplay of genetic factors that predispose to SLE and a range of environmental triggers (Fig. 1). This complex interaction culminates in the generation of autoantibodies with pathogenic potential. These lupus-associated autoantibodies collaborate with both innate and adaptive immune responses, promoting the accumulation of immune complexes across diverse tissues and organs. Such deposition initiates episodes of inflammation, both acute and chronic, ultimately inflicting damage upon the affected organs [1].

### Immune dysregulation

**Fig. 2:**
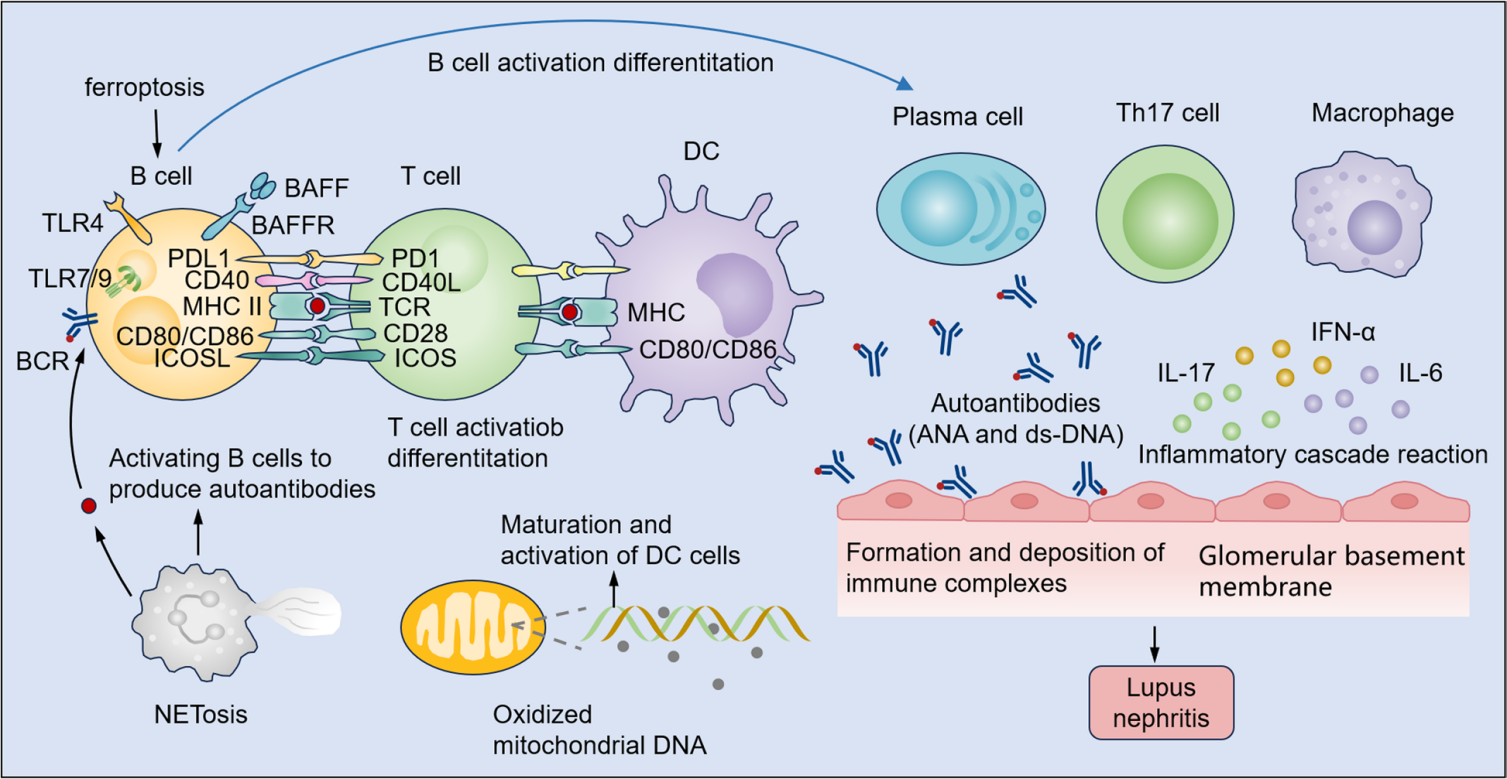
Immunological interplay driving SLE pathogenesis. In SLE, the pathogenesis of lupus nephritis involves an intricate interplay among B cells, T cells, DCs, and other immune components. Key aspects include: (1) Excessive production of autoantibodies by aberrant B cells leads to the formation of immune complexes, which deposit in the kidneys, activating the complement system and inciting inflammatory responses. (2) Autoreactive B cells present self-antigens to T cells, perpetuating immune dysregulation and fostering the release of pro-inflammatory mediators. (3) Dendritic cells exhibit aberrant recognition and presentation of self-antigens, thereby initiating and propagating autoimmune responses against renal tissue. (4) B cell differentiation into autoantibody-secreting plasma cells is dysregulated, resulting in the sustained production of nephritogenic autoantibodies. Elucidating these pathogenic mechanisms is pivotal for the development of novel therapeutic strategies aimed at halting or reversing the progression of lupus nephritis

A pivotal factor in the pathogenesis of SLE is the immune response’s dysregulation, which encompasses both innate and adaptive immune systems. This disruption is instrumental in the development and progression of disease. Evidence suggests that the innate immune system’s dysfunction, which includes reduced neutrophil phagocytic capacity and increased oxidative stress, is accompanied by a buildup of dendritic cells (DCs) at inflammatory locations [7], and defects or mutations in the complement system [8], are linked to SLE. Additionally, the compromised function of adaptive immunity, such as augmented B cell activity, failure in the clearance of autoreactive B cells [9], and the overactivation of T cells, can lead to an upsurge in autoantibody production [9, 10]. These discoveries highlight the complex interaction between innate and adaptive immune responses in the development of SLE (Fig. 2). Studies have indicated that elevated levels of type I interferons (IFNs) are detectable in SLE and are associated with the severity of the disease [11]. We will examine the mechanisms of aberrant immune cell activation, the role of type I interferons and the breakdown of immune tolerance.

#### Aberrant immune cell activation

In SLE, pathogenesis is driven by the abnormal activation of both adaptive and innate immune cells. Adaptive immune cells, including T lymphocytes, B lymphocytes, and plasma cells, play a central role in the disease’s development. T cells may suffer a loss of self-tolerance, resulting in the stimulation of B cells by autoreactive cells. This activation leads to an overproduction of autoantibodies by B cells, which then form immune complexes. These complexes accumulate in tissues, initiating an inflammatory response. Innate immune cells, such as neutrophils, monocyte-macrophages, natural killer cells and DCs, also contribute significantly to SLE pathogenesis [12]. This interplay between adaptive and innate immune cells, along with the overproduction of pro-inflammatory cytokines and the disruption of regulatory mechanisms, culminates in chronic inflammation and multi-organ damage characteristic of SLE.

##### B cells

B lymphocytes are distinguished by the presence of the B-cell receptor (BCR) on their cell membrane, a receptor that is naturally designed for the detection of pathogens and the subsequent synthesis of specific antibodies [13]. Throughout B-cell maturation, there is potential for the emergence of autoreactive B cells. Typically, the immune system employs tolerance mechanisms, such as clonal deletion and peripheral anergy induction, to regulate the development of these potentially harmful cells. However, these regulatory processes are not infallible and can at times be circumvented. This may result in the unintended expansion and stimulation of autoreactive B cells, potentially instigating the onset of autoimmune diseases [14–17]. The survival and proliferation of B cells after maturation, particularly those with the capacity for autoreactivity, are significantly regulated by various soluble factors. A key player among these factors is B-cell activating factor (BAFF), also known as B lymphocyte stimulator (BLys) [18, 19]. The range of autoantibodies generated by B cells with autoreactive properties is primarily targeted against antigens found in the cell nucleus. Toll-like receptors (TLRs), particularly TLR7 and TLR9, play a pivotal role in the genesis of these autoantibodies. In SLE, the abnormal stimulation of TLR9 and TLR7 has been shown to markedly increase the generation of autoantibodies that target double-stranded DNA (dsDNA) and RNA-related autoantigens, respectively [20–23]. Long-lived plasma cells (LLPCs), resulting from the terminal differentiation of B cells, are a crucial source of autoantibody generation in SLE. Short-lived plasmablasts, upon interaction with CD4 + T cells within the germinal centers of lymph nodes, have been observed to evolve into high-affinity plasma cells. These cells then migrate to specialized niches within the bone marrow where they are shielded from external influences, enabling them to persist over extended periods and continuously produce autoantibodies [24]. The autonomous emergence of germinal centers, promoting the maturation of LLPCs, is a characteristic evident in both mouse models and human cases of SLE. This phenomenon suggests a significant link to the etiology of autoantibody generation [25]. Notably, in SLE, B lymphocytes have been demonstrated to act as antigen-presenting cells (APCs) to T lymphocytes with autoreactive potential, as observed in mouse models [26, 27].

##### T cells

Autoreactive T cells are pivotal in the development of SLE. T-helper 1 (Th1) cells are particularly influential in SLE pathogenesis, as they foster oxidative stress linked to the production of interferon-gamma (IFNγ) [28]. Inversely, a reduction in Th2 cells, which produce IL-4, in the peripheral blood of SLE patients suggests they may have a protective role, with disease activity possibly correlated to an increased ratio of IFNγ to IL-4 [29]. Furthermore, T-helper 17 (Th17) cells play a role in the pathogenesis of SLE, acting as the main producers of IL-17, a highly inflammatory cytokine. This cytokine promotes the recruitment of neutrophils, triggers the activation of the innate immune system, and enhances the functionality of B lymphocytes [30]. Studies have indicated that IL-17 levels are correlated with the SLE Disease Activity Index (SLEDAI) in patients with lupus nephritis [31, 32].

Regulatory T cells (Tregs) play a vital role in preserving peripheral tolerance towards self-antigens. In SLE, there is evidence of both quantitative and qualitative disparities in Tregs, yet research has yielded inconsistent findings, leaving their precise role in SLE yet to be fully defined. Nonetheless, some investigations suggest that Tregs, owing to their suppressive effect on effector T lymphocytes, could be instrumental in foundational cellular therapies for SLE [33–35]. T-follicular helper (Tfh) cells, found in both germinal centers and extrafollicular areas, are associated with the expansion of autoreactive B-cell clones. This association has been observed in both mouse models and in individuals with SLE [36]. These cells have been observed to congregate with B cells in renal tissues, mirroring their aggregation in germinal centers during lupus nephritis [37]. Altogether, these findings highlight the crucial role of the interaction between CD4 + T cells and B cells in the development of autoimmune conditions. This interaction significantly promotes the development and maintenance of autoreactive B cells, as well as their differentiation into plasma cells that produce autoantibodies.

CD8 + T cells contribute to the immune-mediated pathology observed in SLE. Researches have revealed that CD8 T lymphocytes in the peripheral blood of SLE patients exhibit functional deficits, such as an impaired ability to lyse target cells and a reduction in the synthesis of granzymes and perforins [38]. A diminished circulating phenotype of CD8 T lymphocytes in SLE patients has been correlated with a reduced frequency of disease flares [39]. However, qualitative irregularities in CD8 + T lymphocytes also predispose SLE patients to infections, a vulnerability that can be exacerbated by immunosuppressive therapy [40]. Lastly, γδ-T lymphocytes have been observed at higher frequencies in SLE patients compared to healthy controls, suggesting their involvement in autoimmune responses [41, 42].

##### Neutrophils

In SLE, neutrophil dysfunction has been observed at multiple levels. Initially, neutrophils exhibit a diminished capacity for phagocytosis and a failure to clear apoptotic cells, which are a source of self-antigens typically sequestered from the immune system [43–45]. Additionally, neutrophils have been shown to produce type-I IFNs without the need for TLR stimulation, which can lead to abnormal B-cell development within the bone marrow of SLE patients [46, 47]. A particular subtype of neutrophils, referred to as low-density granulocytes (LDG), is commonly found in higher numbers in the peripheral circulation of SLE patients. There is a correlation between these LDG cells and the presence of an interferon (IFN) signature, as well as the overall disease severity [48–50]. , and they can activate CD4 + T-cells, prompting them to generate IFNγ and TNFα [48].

In SLE, low-density granulocytes (LDGs) are distinguished by their heightened capacity to produce neutrophil extracellular traps (NETs), particularly during the process of NETosis, which occurs as they undergo apoptosis [51]. NETs are composed of decondensed nucleic acids and expelled chromatin, and their formation can elicit autoreactive immune reactions against nucleic acid antigens [52]. Additionally, neutrophils are known for generating reactive oxygen species (ROS), which, while typically crucial for pathogen elimination, can inflict endothelial damage in SLE [52]. Various genetic polymorphisms associated with neutrophil dysregulation and increased NET formation have been identified in SLE patients [53–55]. Furthermore, neutrophils from SLE patients with STAT3 function impairments have been observed to form NETs more readily compared to those from healthy individuals [56]. Excessive NET formation coupled with impaired clearance may also result in heightened inflammasome activation in macrophages, thereby exacerbating the inflammatory response [57]. In aggregate, these findings underscore the significant immunostimulatory role of neutrophils in SLE through NET formation, which substantially contributes to immune dysregulation and subsequent tissue injury.

##### DCs

DCs, first identified by Ralph Steinman in 1973, serve as the principal antigen-presenting cells. They are crucial in the context of SLE, as they bridge the innate and adaptive immune responses [58]. DCs function as key regulators in SLE-related autoimmunity, embodying both stimulatory and suppressive roles. They possess the capacity to discern self and non-self-antigens through pattern recognition receptors (PRRs), initiating maturation and the secretion of inflammatory cytokines and type I interferons (IFN-I). This process can lead to the activation of autoreactive T and B cells, which contribute to the inflammatory pathology of SLE. Conversely, DCs with tolerogenic capabilities can foster immune tolerance, either by promoting the function and development of regulatory T or B cells or by inhibiting the activation of these cells. This duality aids in curbing pathological inflammation and autoimmunity [59, 60].

Chronic exposure to autoantigens leads to the maturation and activation of conventional dendritic cells (cDCs). Once activated, cDCs facilitate the differentiation of effector T (Teff) cells and intensify B cell activation and auto-antibody production within the germinal center (GC). This sequence of events culminates in the disruption of immune tolerance and perpetuates tissue inflammation. In SLE, DCs exhibit heightened maturation markers, including elevated levels of costimulatory molecules like CD86, and they secrete proinflammatory cytokines, which contribute to disease pathogenesis [61, 62]. Furthermore, follicular dendritic cells (FDCs) are capable of capturing and retaining self-antigens through CD21, which triggers TLR7/interferon regulatory factor 5 (IRF5)-mediated IFN-I production. This process enhances GC reactions and the generation of pathogenic auto-antibodies in lupus pathogenesis [63]. Plasmacytoid dendritic cells (pDCs), which originate from the lymphoid lineage, are distinguished by their capacity to generate substantial amounts of type-I IFNs, thereby significantly contributing to the development of SLE [64–66].

#### Aberrant type I IFN activation

A significant discovery in the pathogenesis of SLE that has paved the way for innovative drug development is the identification of a heightened type-I interferon (IFN) signature among SLE patients [67]. In SLE, it is primarily the type-I IFN, particularly IFNα and IFNβ, that is implicated in disease pathogenesis. Type-I IFNs are initiated by the stimulation of PRRs, including TLRs, retinoic acid-inducible gene I (RIG-I), and melanoma differentiation-associated protein 5 (MDA5). These receptors are activated by nucleic acids or bacterial substances such as lipopolysaccharides and peptidoglycan [68]. While various cell types can produce type-I IFN [69], pDCs are particularly notable for their high synthesis of this cytokine, as previously mentioned [70–72]. Type-I IFNs engage with the interferon-alpha receptor (IFNAR), a heterodimeric complex that initiates intracellular signaling cascades via Janus kinase 1 (JAK1) and tyrosine kinase 2 (TYK2). Activation of these kinases leads to the phosphorylation of signal transducers and activators of transcription 1 and 2 (STAT 1 and STAT 2), which are key transcription factors. Once phosphorylated, STAT proteins complex with IRF7 and IRF9 to form the ISGF3 complex. This complex migrates into the nucleus, triggering the transcription of genes that harbor IFN-sensitive response elements (ISREs). The proteins encoded by these genes contribute to the amplification of inflammatory responses [73, 74].

Early experimental studies using animal models demonstrated that administration of type-I IFNs could induce autoantibody production and lead to organ damage [75]. The first indications that type one interferon might play a pivotal part in SLE development in humans came from observations that patients treated with IFNα for hepatitis C [76] or malignancies [77, 78] could develop antinuclear antibodies and, in some cases, lupus-like syndromes. These situations often resolved upon cessation of IFNα therapy [78, 79]. Further studies have discovered that gene polymorphisms in the type-I IFN signaling pathway, including the IRF gene family, are substantial genetic risk factors for SLE [80–82]. Additionally, polymorphisms have been found in TYK2, STAT3, and STAT4 genes [56, 83], further underscoring the importance of this pathway in the disease’s etiology. The IFN signature is increasingly recognized as a potential biomarker, guiding the targeted use of emerging anti-IFN therapeutics for precision treatment.

#### Loss of tolerance

The collapse of immune tolerance plays a pivotal role in the development of autoimmune conditions; however, the precise mechanisms driving this breakdown remain to be fully elucidated. Immunological tolerance is divided into two main types: central and peripheral. Central tolerance occurs in the thymus for T cells and in the bone marrow for B cells, serving as the principal process through which the immune system learns to differentiate self from non-self-antigens. Peripheral tolerance, on the other hand, is established in tissues and lymph nodes following the maturation of lymphocytes. It serves to regulate self-reactive immune cells and to prevent excessive immune reactions to environmental stimuli. When tolerance is compromised, it can lead to the development of autoimmune conditions, including SLE.

### Genetic predisposition

The origins and mechanisms behind autoimmune diseases are exceedingly intricate. A combination of epigenetic changes and genetic susceptibility contributes to the failure of immunological tolerance, leading to autoimmunity. In this review, we will delve into how epigenetic and genetic factors influence the collapse of tolerance in autoimmune conditions.

#### Polygenic inheritance

The genetic predisposition to lupus in humans is firmly established, as evidenced by the markedly higher disease concordance rates among monozygotic twins (ranging from 25 to 57%) compared to dizygotic twins (2–9%) [84]. Among the genetic regions consistently implicated in SLE are those located on chromosome 1. The 1q23 linkage interval includes the genes for Fcγ receptors, FCGR2A and FCGR3A. Variations in these receptors, which have different affinities for IgG and its subclasses, can result in inefficient clearance of immune complexes, potentially causing their accumulation in the blood vessels and kidneys [85]. Other SLE-associated genes on chromosome 1 encompass PTPN22 [86], IL10 [87], and C1Q [88].

The major histocompatibility complex (MHC) genes, as well as components of the complement system such as C2 and C4, and tumor necrosis factor (TNF) genes TNFα and TNFβ, are located on chromosome 6. Polymorphisms within these genes have been shown to confer susceptibility to SLE [89]. The programmed cell death 1 gene (PDCD1), which is raising in T cells to suppress T cell receptor (TCR) signaling and T and B cell survival, harbors a single nucleotide polymorphism (SNP) within an intron that is associated with SLE risk in Europeans. This SNP impacts the binding location of the runt-related transcription factor 1 (RUNX1), implying its role in SLE pathogenesis [90]. Additionally, CTLA4, a negative costimulatory molecule that curbs T cell activation and potentially limits T cell reactivity during inflammatory responses, exhibits genetic variability that has been correlated with an increased risk of developing SLE [90, 91].

#### Epigenetic modifications

Epigenetic alterations encompass heritable changes that occur without altering the underlying DNA sequence, manifesting at the levels of DNA, RNA, or proteins [92]. Epigenetic mechanisms play a significant role in SLE and are capable of modulating gene expression through several pathways, including DNA methylation, histone post-translational modifications, and the action of microRNAs [93]. In SLE, the global hypomethylation of T cell DNA represents a characteristic epigenetic modification. This alteration results in increased gene transcription, which is closely linked to the severity of the disease [94]. When epigenetic regulation becomes disrupted, it can lead to misexpression of genes and dysfunction in immune cells, potentially triggering autoimmune reactions.

In terms of adaptive immunity, these epigenetic disruptions are particularly pivotal in the activation of T cells within the SLE context. The activation of Tfh cells is known to foster the generation of autoantibodies. Furthermore, epigenetic dysregulation is implicated in the suppression of Treg differentiation, thereby amplifying the pool of autoreactive lymphocytes [95].As for the innate immune response, epigenetic alterations are shown to enhance the production of IFNs, which in turn, trigger the expression of genes regulated by type I IFNs in SLE. Consequently, these findings have shed light on the intricate pathogenic mechanisms underlying SLE.

### Environmental factors

Prominent environmental factors known to precipitate SLE include viral infections, certain medications, tobacco use, and exposure to ultraviolet (UV) radiation [96, 97].

#### Infections

Infections, encompassing viruses, bacteria, parasites, and fungi, are significant environmental contributors to the genesis of autoimmune conditions. Notably, viral infections have been particularly implicated in the etiology of SLE [98]. Human endogenous retroviruses (HERVs) are considered to predispose individuals to SLE due to genetic factors, while the Epstein-Barr virus (EBV) is recognized as an environmental trigger that may initiate SLE. It is hypothesized that B-cells infected with EBV might evade apoptosis, leading to their activation, proliferation, and the generation of autoantibodies. These autoreactive B-cells are implicated in the tissue damage characteristic of SLE.

EBV is also posited to play a part in the activation of the innate immune system and B cell maturation, potentially inciting the generation of autoantibodies that target amino acid sequences common to both self-proteins and those encoded by EBV. The virus’s small RNAs are known to provoke immune responses by inducing the production of IFNs. This occurs through binding to the dsRNA-dependent protein kinase, thereby triggering a TLR-independent signaling pathway. Furthermore, antibodies directed against the EBV-encoded Epstein-Barr nuclear antigen 1 (EBNA1) may exhibit cross-reactivity with double-stranded DNA, indicating a possible link between EBV infection and the induction of autoimmune responses [99]. While the exact molecular underpinnings of this cross-reactivity remain elusive, it is speculated that shared conformational epitopes between DNA and EBNA1 may be the basis for this phenomenon.

#### Ultraviolet radiation

UV radiation, encompassing both UVA and UVB wavelengths, plays a significant role in SLE pathogenesis. This radiation primarily induces apoptosis in keratinocytes through the producing of reactive oxygen species (ROS) and the infliction of DNA damage. UVB radiation-induced DNA damage triggers intracellular nucleic acid sensors and PRRs, including stimulator of interferon gene (STING). This process leads to the production of type-1 IFNs [100]. Under conditions of elevated circulating type-1 IFN, Langerhans cells and keratinocytes that have been exposed to UV radiation express high levels of pro-inflammatory cytokines including IL-6, IL-1β, and TNF-α [101, 102].This cytokine-rich environment stimulates the T-cells recruitment, establishing a self-sustaining inflammatory loop that activates the adaptive immune system [103, 104].

UV radiation-induced apoptosis can lead to the relocation of intracellular antigens such as Ro59, Ro60, and the interferon-inducible protein 16 (IFI16) to the cytoplasmic membrane [105, 106]. In lupus-prone individuals, the clearance of apoptotic cells is typically less efficient, which extends the immune system’s exposure to these antigens [107–109]. This prolonged exposure can stimulate the generation of autoreactive CD8 + T-cells and the production of autoantibodies that target these sequestered antigens [104]. Antibodies against Ro60 and Ro59, known as anti-Ro antibodies, can intensify inflammation by interfering with the regular functions of these proteins [110]. The Ro60 protein is involved in the removal of incorrectly folded non-coding RNAs, which can cause inflammation if they build up inside cells. In parallel, the Ro59 protein interacts with interferon regulatory factors (IRFs), thus affecting the cell’s reaction to interferon signaling.

### Immune complexes

Immune complexes (ICs) are a common characteristic in immune-mediated responses that protect the host against diseases. However, under specific conditions, they can become detrimental to health. SLE serves as a recognized exemplar of immune complex involvement in autoimmune disorders. It is plausible that the various mechanisms of IC formation and their subsequent effects contribute significantly to the development of lupus nephritis, cutaneous involvement, and other clinical features of SLE. The assessment of immune complexes plays a practical role in disease monitoring and in the evaluation of certain therapeutic interventions. SLE presents as chronic or paroxysmal inflammation in multiple organ systems associated with the presence of circulating and tissue-deposited Immune complex, these Immune complexes stimulate white blood cells through the FC gamma receptor (FcγR) and subsequently inflammation.

In SLE, the buildup of immune complexes and autoantibodies can trigger inflammation and damage in specific organs [111]. Although SLE can impact any organ, it frequently targets kidneys, joints, central nervous system, hematologic system, and skin. Mucocutaneous manifestations are particularly prevalent, with up to 75% of patients experiencing them at some point and 25% at diagnosis. The most typical cutaneous manifestation is a malar rash resembling a butterfly on the nose and cheeks, accompanied by photosensitivity, oral/nasal ulcers, scarring discoid lesions and non-scarring alopecia [98].

## Targeted therapies of SLE

SLE is a chronic autoimmune disease, distinguished by its impact on multiple organs and the diverse clinical presentations and disease severity it exhibits [112]. For many years, the treatment of SLE has primarily involved non-specific approaches, including cytotoxic and immunosuppressive drugs [4], antimalarial agents [113], non-steroidal anti-inflammatory drugs (NSAIDs), and glucocorticoids. However, in more recent times, the therapeutic landscape for SLE has evolved to incorporate more precise strategies, such as cellular therapies and precision medicine [114].

### Traditional therapies of SLE

SLE encompasses a spectrum of clinical presentations, reflecting its intricate autoimmune nature. Although current treatment strategies can effectively reduce the severity of the disease, they do not provide a cure and often come with side effects. For milder cases of SLE, initial treatment typically includes nonsteroidal NSAIDs and antimalarial medications. (Table 1). For more severe cases, particularly those with organ involvement, a range of glucocorticoids and immunosuppressive therapies, including azathioprine, mycophenolate mofetil, cyclophosphamide, cyclosporine, and methotrexate, are employed [115].

**Table 1:** Traditional therapies in SLE

Type of agent	Agent	Mechanisms	Risks
Cytotoxic and immunosuppressive therapies	Methotrexate	Inhibiting the activity of the enzyme aminoimidazole, carboxamide, ribonucleotide, transformylase	Gastrointestinal events, Development of pulmonary tuberculosis
	Azathioprine	Suppress indnucleic acid synthesis and impacting immune responses	Gastrointestinal intolerance, Bone marrow suppression
	Cyclophos -phamide	Crosslinking DNA and proteins associated with DNA	Infection, Gonadal toxicity, Myelosuppression, Carcinogenic effect, Bladder toxicity
	Mycophenolate mofetil	Inhibitor of inosine monophosphate dehydrogenase	Fetal malformation and abortion
	Sirolimus	Inhibitor of the mTOR signaling pathway	Hematological abnormalities, mucocutaneous abnormalities, dyslipidemia
Non-Steroidal Anti-Inflammatory Drugs (NSAIDs)	Celecoxib rofecoxib	Inhibitor of cyclooxygenase enzymes	Gastrointestinal and cardiovascular complications
Antimalarials	Hydroxy -chloroquine	causing interference with cyclic GMP-AMP (cGAMP) synthase	HCQ-related retinopathy, Gastrointestinal intolerance, Skin pigmentation, Antimalarial-induced myocardiopathy, Neuromyopathy, hypoglycemia, tinnitus, vertigo, dizziness
Glucocorticoids (GCs)	GCs	Binding the cytosolic-GC receptor (cGR) and being mediated by the GC-cGR complex, by membrane-bound GR (mGR) or by nonspecific interactions with cellular membranes	Lacking evidence supporting the long-term stability benefits, Increased risk of infections, Osteoporosis, fractures and weakness, Hyperglycemia/Diabetes, Cushing syndrome, Cardiovascular disease, Glaucoma, Cataracts

#### Cytotoxic and immunosuppressive therapies

The use of cytotoxic and immunosuppressive medications is typically reserved for individuals with severe SLE manifestations. This class of drugs encompasses methotrexate, azathioprine, cyclophosphamide, and mycophenolate mofetil (MMF). Less commonly, cyclosporine and leflunomide are also utilized. Most evidence supporting the use of these agents is derived from their application in lupus nephritis. Except for azathioprine, these medications are generally contraindicated during pregnancy.

Methotrexate (MTX) functions by inhibiting the activity of the enzyme aminoimidazole carboxamide ribonucleotide transformylase, which results in an increase in adenosine levels. This elevation in adenosine contributes to anti-inflammatory properties and can suppress the activity of neutrophils [116]. This mechanism is believed to be, at least partially, responsible for its therapeutic efficacy in the treatment of autoimmune conditions [116]. In SLE, methotrexate has shown effectiveness in patients exhibiting articular or dermatological symptoms, facilitating a reduction in steroid requirements and modestly attenuating disease activity [117, 118].

Azathioprine (AZA), a purine analog, suppresses nucleic acid synthesis and impacts both cellular and humoral immune responses. It exhibits efficacy in SLE patients presenting with arthritis, serositis, and mucocutaneous involvement [119]. Frequently utilized as a means to reduce steroid dependence, AZA has demonstrated effectiveness in sustaining remission of the disease [119].

Cyclophosphamide (CYC), a nitrogen mustard-derived alkylating and cytotoxic agent, functions by crosslinking DNA and proteins associated with DNA, thereby impeding DNA replication and inducing cellular death. In combination with high-dose glucocorticoids, CYC has been a cornerstone therapy for severe SLE that poses a threat to vital organs, encompassing lupus nephritis, neuropsychiatric manifestations of lupus, and severe systemic vasculitis [119, 120].

MMF, a prodrug that converts to mycophenolic acid (MPA), is a significant immunosuppressive agent employed in the treatment of SLE. MMF’s mechanism of action involves the inhibition of inosine monophosphate dehydrogenase, a key enzyme in the de novo pathway of guanosine nucleotide synthesis9. Furthermore, MPA is known to modulate dendritic cell subsets, thereby disrupting the detrimental cycle of autoimmune reactions [121]. At present, MMF is extensively utilized for both induction and maintenance therapy in LN [122, 123]. It has also been implicated in the management of patients with extrarenal manifestations of SLE [124, 125]. A 24-month, multicenter, randomized clinical trial [126] has demonstrated that MMF outperforms azathioprine in the treatment of SLE, including the prevention of disease relapses. The results from a randomized clinical trial indicate that MMF might decrease the frequency of severe disease exacerbations and reduce the likelihood of developing LN in individuals with newly diagnosed SLE who have elevated levels of anti-dsDNA antibodies but no significant involvement of major organs [127].

Sirolimus, recognized alternatively as rapamycin, represents an immunosuppressive agent that is being explored for its therapeutic potential in SLE. Its mechanism involves the inhibition of the mTOR signaling pathway, which in turn reduces immune cell activity and mitigates inflammatory and autoimmune reactions [128, 129]. Clinical experiences have shown that sirolimus can enhance the population of Tregs, ameliorate disease activity in patients with active SLE, and curb the production of IL-17 and IL-4, all without raising safety issues [130]. Moreover, sirolimus has demonstrated efficacy comparable to tacrolimus and MMF in the treatment of SLE or LN patients. It has even shown superior serological improvements and a greater reduction in glucocorticoid use, as evidenced by real-world data from the Research group (CSTAR) cohort studies and Chinese SLE Treatment. Sirolimus has been well-tolerated among SLE patients [131]. Nonetheless, its use must be approached with caution due to immune suppression and potential side effects, requiring vigilant clinical monitoring. Further research is essential to substantiate sirolimus’s therapeutic efficacy in SLE.

#### Non-steroidal anti-inflammatory drugs (NSAIDs)

NSAIDs are typically effective for alleviating mild SLE symptoms, including arthralgia, musculoskeletal issues, fever, headaches, and mild serositis [119]. These medications function as inhibitors of cyclooxygenase enzymes, specifically cyclooxygenase-2 (Cox-2) and cyclooxygenase-1 (Cox-1). The advent of selective Cox-2 inhibitors like celecoxib and rofecoxib has allowed for a categorization of NSAIDs into non-selective Cox inhibitors, preferential Cox-1 inhibitors, and selective Cox-2 blockers. Among NSAIDs, naproxen is considered to possess a relatively higher cardiovascular safety profile compared to other members of its class [132]. Experts advocate for the use of NSAIDs with a high degree of selectivity for COX-2, such as celecoxib, to minimize adverse effects. In contrast, NSAIDs with lower selectivity, like piroxicam and ketorolac, are generally not preferred. A retrospective study of 50 patients with mild SLE demonstrated celecoxib’s efficacy and safety, with reduced gastrointestinal toxicity compared to other NSAIDs. However, due to its sulfonamide structure, celecoxib may not be suitable for patients with allergies to this chemical group [132, 133].

#### Antimalarials

Antimalarial medications are particularly effective in alleviating SLE constitutional symptoms, such as fatigue and fever, as well as musculoskeletal, dermatological, and mild pleuritic issues [113]. Research indicates that these drugs are instrumental in maintaining remission in SLE, averting severe disease relapses, and shielding the kidneys and central nervous system from considerable harm [134, 135]. They also serve to decrease the necessary dosage of prednisone. Additional advantages of antimalarial therapy include a diminished risk of thromboembolic events in individuals with antiphospholipid antibodies [9], improved glycemic management and insulin sensitivity in SLE patients, thereby reducing the likelihood of diabetes development [136]. These medications also enhance lipid profiles by curbing cholesterol synthesis and upregulating LDL receptor activity [136, 137], bolster bone density [138], and lower the risk of cancer [139].

Hydroxychloroquine (HCQ) stands out as the predominant antimalarial agent for treating rheumatic conditions in the United States, given its reduced risk of retinal toxicity compared to chloroquine. Moreover, hydroxychloroquine is considered relatively safe for use during pregnancy, making it a favorable choice in certain clinical scenarios [119]. Its use, either as a standalone treatment or in conjunction with steroids and immunosuppressive agents, has become integral to the management of SLE, enhancing patient survival by mitigating lupus flares and preventing the progression of organ damage. The 2019 revisions to the EULAR guidelines for managing SLE advocate for the administration of HCQ in all lupus patients, barring contraindications, with a high level of evidence and recommendation grade (1b, grade A) [4]. A prospective cohort study has underscored HCQ’s efficacy in alleviating classical manifestations of SLE, such as dermatological issues and arthralgia, through its anti-inflammatory properties and the reduction of autoantibody levels [140].

#### Glucocorticoids (GCs)

GCs are potent anti-inflammatory agents that swiftly mitigate inflammation and modulate both the innate and adaptive arms of the immune system, leading to an improvement in SLE symptoms [141]. The dosage of glucocorticoids is tailored to the severity of the disease and the extent of organ involvement [142–144]. For six decades, glucocorticoids have served as a cornerstone in the management of SLE. An escalation in glucocorticoid dosing is frequently implemented to address small spikes in disease activity [145]. However, it is important to note that there is a lack of evidence supporting the long-term stability benefits of augmented steroid therapy [146]. Oral glucocorticoids at a low dosage can be equally effective in managing systemic active extrarenal disease as higher doses, such as 30 mg/day or more [147]. This suggests that the standard dosage could be reduced in routine practice. Consequently, during periods of disease exacerbation, it is recommended to utilize the minimum effective dose of glucocorticoids, with a defined plan for treatment duration and clear therapeutic objectives.

### Emerging targeted therapies

Emerging targeted therapies for SLE are designed to address specific components of the immune system that contribute to the disease’s pathogenesis. These therapies aim to provide more precise treatment with fewer side effects compared to traditional broad immunosuppressive approaches. Here’s an overview of some of the targeted therapies that are currently in development or under investigation for SLE.

#### Cellular therapies

Cell therapy is a medical technique that uses cells as a therapeutic tool. It has shown great potential in the treatment of many diseases, including SLE. In the treatment of SLE, cell therapy mainly targets B cells and T cells, because these cells play a key role in the pathogenesis of the disease. Here, we outline the targeting of B and T cells for the treatment of SLE.

##### Targeting B cells as a therapeutic strategy for SLE

**Fig. 3:**
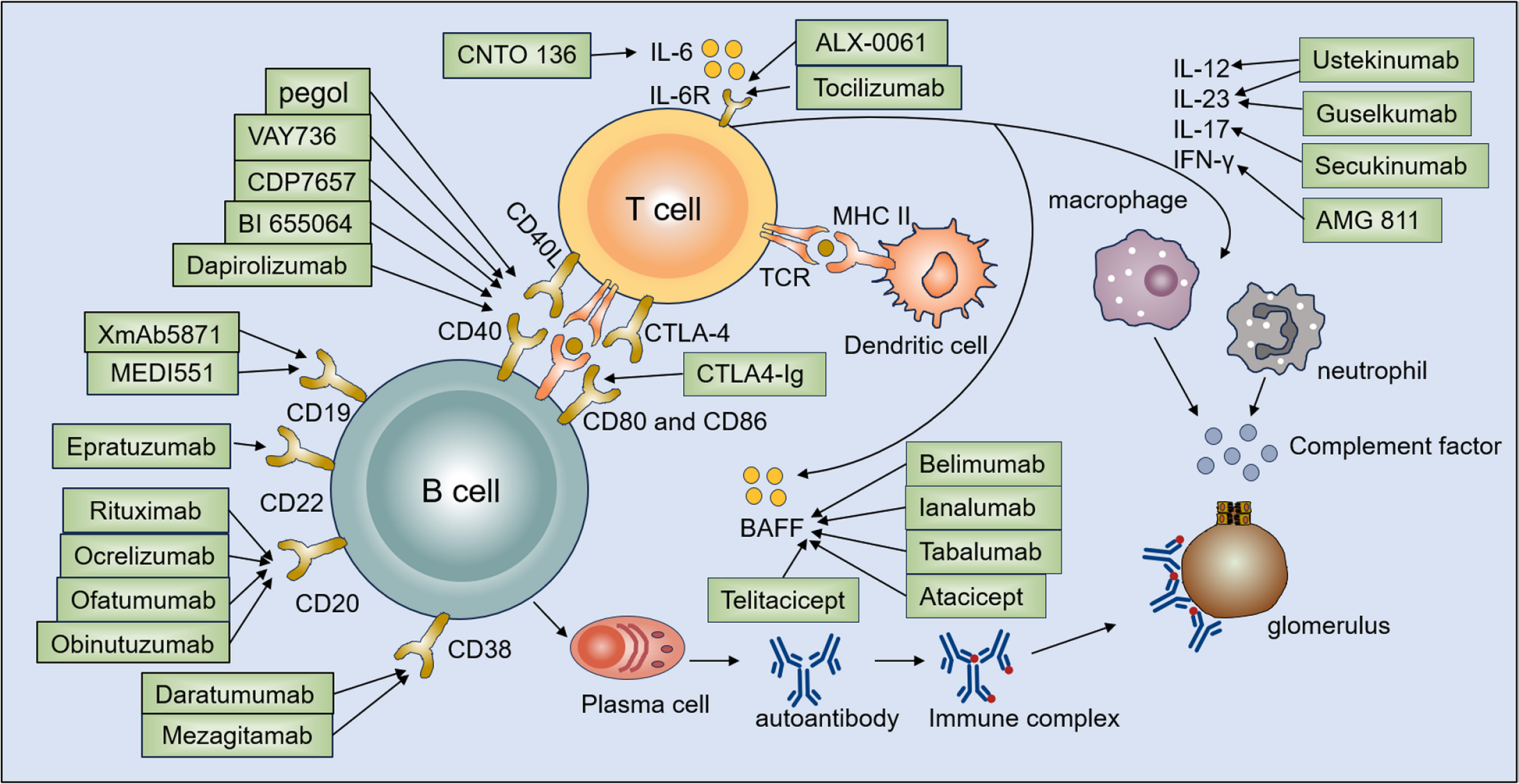
Targeted therapies of SLE based on B and T cells. The therapeutic strategies aimed at B cells include targeting B-cell surface receptors (CD10, CD20, CD22, CD38, CD40) and targeting B-cell related cytokines (BAFF). The therapeutic strategies aimed at T cells include co-stimulation blockade (CD40–CD40L, CD80/86–CD28, ICOS–ICOSL) and cytokine blockade (IL-6, IL-12, IL-17, IL-23, IFN-γ)

The therapeutic strategies aimed at the B cell compartment can be broadly outlined as targets B cell surface receptors and targeting B cell related cytokines (Table 2; Fig. 3).

**Table 2:** B-cell targeted therapies in SLE

Type of agent	Agent	Target	Stage of development	Reference
Targets B cell Surface Receptor	Rituximab	CD20	phase III trial	NCT03312907 [148]
	Ocrelizumab	CD20	phase III trials	[149] NCT00626197[150]
	Obinutuzumab	CD20	phase II trial phase III trials	NCT02550652[151] NCT04702256 NCT04963296
	Ofatumumab	CD20		[152]
	XmAb5871	CD19	phase II trial	NCT02725515
	MEDI551	CD19	Phas IIa trail	NCT06570798
	Epratuzumab	CD22	phase IIb trail phase III trial	[153] [154]
	Daratumumab	CD38	phase II trials	NCT04810754 NCT04868838
	Mezagitamab	CD38	phase Ib trial	NCT03724916
Targeting B Cell Related Cytokines	Belimumab	BAFF	phase III trials phase IV trail	[155] [156] NCT04515719
	Ianalumab	BAFF	phase III trials phase II trial	NCT05624749 NCT06133972 NCT03656562
	Tabalumab	BAFF	phase III trials	[157] [158]
	Atacicept	BAFF	phase II/III trial phase IIb trail	NCT00624338[159] [160]
	Telitacicept	BAFF	phase III trials phase I trials phase II trials phase IV trials	NCT04082416 NCT06456567 NCT05339217 NCT05306574 NCT05247203 NCT05687526 NCT05929248 NCT05680480 NCT05899907 NCT05666336

CD20 is consistently expressed throughout the development of B cells, except for the fully differentiated plasma cells and earliest pre-B cells. Despite lacking a known ligand, CD20 functions not only as a significant clinical marker for identifying B cells but also as a target for therapeutic intervention [148]. CD20, potentially modulates calcium-dependent signaling pathways through indirect regulation of B cell receptors, thereby governing B cell proliferation and differentiation [149].

Rituximab, a chimeric monoclonal antibody (mAb) against CD20, its therapeutic impact on SLE is still a subject of debate [150–152]. However, Numerous observational studies and routine clinical applications have evidenced the effectiveness of rituximab in managing refractory lupus nephritis and severe non-renal SLE manifestations, encompassing hematological disorders, severe joint involvement, neuropsychiatric disease, and dermatological conditions [153–156]. An investigation is designed to evaluate if the conjunctional use of belimumab with a solitary course of rituximab can augment the efficacy of belimumab monotherapy, leading to enhanced clinical outcomes alongside a beneficial safety profile. This will be accomplished through a comparative analysis of subjects allocated to concurrent treatment with belimumab and rituximab versus those receiving belimumab coupled with a rituximab-placebo (NCT03312907). Encouraged by the positive findings from combined therapeutic strategies, a multicenter phase III clinical trial is now in progress to further assess the efficacy of this novel treatment method for patients with severe SLE [157].

Ocrelizumab, a recombinant humanized mAb, is designed to target and deplete CD20 + B cells from the peripheral circulation. It has been the focus of two clinical trials addressing SLE [152, 158]. The phase III BEGIN study, which focused on non-renal SLE, was prematurely halted due to the sponsor’s decision to discontinue this line of investigation [152]. Conversely, the BELONG study, a randomized, double-blind, phase III trial (NCT00626197), assessed the efficacy of ocrelizumab in conjunction with cyclophosphamide or mycophenolate mofetil in patients with lupus nephritis. Interim results at the 32-week mark indicated renal response rates of 63% in the ocrelizumab group and 51% in the placebo group, with a notable advantage observed in patients receiving cyclophosphamide as their background treatment. Nonetheless, an elevated occurrence of serious infections in participants treated with background mycophenolate mofetil led to the early cessation of the BELONG study [159].

Obinutuzumab, another fully humanized anti-CD20 mAb, exhibits superior efficacy in inducing B cell cytotoxicity in individuals with rheumatoid arthritis or SLE, having concluded its phase II trial (NCT02550652) [160]. Conversely, obinutuzumab, a type II anti-CD20 antibody containing fucosylated Fc, demonstrates reduced complement-dependent cellular cytotoxicity compared to rituximab, while exhibiting increased antibody-dependent cellular cytotoxicity and heightened phagocytic activity [161]. Clinical data and analyses have informed the development of obinituzumab, which is intended to overcome limitations and resistance associated with rituximab (RTX) treatment [162]. Presently, the phase III REGENCY trial, designed to assess the efficacy and safety of obinutuzumab in patients with ISN/RPS 2003 class III or IV lupus nephritis, is ongoing. Concurrently, a phase III multi-central trial of a randomised, open label, controlled, non-inferiority design is proceeding. The principal purpose of this clinical investigation is to substantiate the non-inferiority of a treatment regimen that eschews supplemental oral corticosteroids yet integrates obinutuzumab in conjunction with MMF, as compared to a traditional regimen that combines oral corticosteroids with MMF. The primary endpoint is the attainment of a complete renal response by the 52nd week of the trial, achieved without resorting to corticosteroids exceeding a predetermined threshold (NCT04702256). An ongoing phase III trial employing a randomized, double-blind, placebo-controlled design, spanning multiple centers, aims to delineate the efficacy and safety of obinutuzumab relative to a placebo in participants diagnosed with autoantibody-positive SLE exhibiting active disease despite receiving standard-of-care therapies(NCT04963296). The findings suggest a new therapeutic strategy for treating SLE patients who have not seen responses to subsequent RTX treatments, by administering obinutuzumab infusions.

Ofatumumab, a fully human mAb that targets CD20, has received approval for clinical utilization. Initially granted approval for the treatment of chronic lymphocytic leukemia (CLL) in 2009, it subsequently gained approval for use in relapsing forms of multiple sclerosis (MS) in 2020. In a clinical study, ofatumumab was administered to 16 patients with severe infusion reactions associated with RTX. Fourteen patients experienced good tolerance to the infusion, resulting in B cell depletion and improved serological markers of disease activity [163]. Moreover, in patients with refractory LN who had adverse reactions to RTX infusions, administering ofatumumab at a dose of 700 mg with a two-week interval led to clinical improvements, notably a decrease in albuminuria levels [164]. Significantly, there have been documented cases of successful treatment with ofatumumab in juvenile SLE patients, suggesting that ofatumumab may provide an alternative therapeutic choice to RTX for SLE [165, 166].

CD19 is a transmembrane protein that is present on B cells throughout their development, until they ultimately differentiate into plasma cells. It physically links with the BCR, enhancing the BCR’s signaling capabilities [167]. In addition to its role in B cell biology, CD19 also functions as a significant clinical marker for B cells and is considered as a possible target for therapeutic interventions [148].

Anti-CD19 mAbs like XmAb5871, now recognized as obexelimab, can be therapeutically effective without necessarily depleting B cells. Instead of causing physical removal, these mAbs inactivate B cells [168]. Obexelimab is an engineered anti-CD19 mAb designed for high-affinity binding to the inhibitory FcγRIIb receptor. It achieves this by simultaneously engaging CD19/BCR along with FcγRIIb on B cells, thereby potently suppressing BCR-induced B cell activation in vitro. This inhibition is mediated through a pathway involving SH2-containing inositol polyphosphate 5-phosphatase (SHIP) [169, 170].

Notably, obexelimab has demonstrated its capacity to modulate in vivo antibody responses, as evidenced by its inhibitory effect on anti-tetanus antibody production in immunodeficient SCID mice transplanted with human peripheral blood mononuclear cells [171].A double-blind, randomized, placebo-controlled trial (NCT02725515) is completed to assess the effectiveness of obexelimab in sustaining remission in SLE following a brief regimen of steroid therapy. The trial’s primary outcome, which was the percentage of patients maintaining disease improvement without deterioration at the 225-day mark, did not achieve its goal. However, the secondary outcome, measuring the duration until disease worsening, was significantly prolonged in the obexelimab group, hinting at potential benefits that merit further exploration. As of now, phase III studies for obexelimab in the treatment of SLE have not been initiated.

It should be highlighted that the anti-CD19 mAb MEDI551, known as inebilizumab, is effective at depleting B cells while specifically preserving regulatory B cells [172]. This selective sparing enhances the appeal of this therapeutic agent. A Phase 2a, open-label, multicenter platform trial (NCT06570798) is designed to evaluate the safety, tolerability, and efficacy of inebilizumab and blinatumomab in individuals with autoimmune diseases. The primary goal of this study is to determine the safety and tolerability profiles of inebilizumab (Subprotocol A) and subcutaneously administered blinatumomab (Subprotocol B) in adults with active, refractory SLE exhibiting nephritis.

In a groundbreaking investigation, researchers generated anti-CD19 chimeric antigen receptor (CAR) T cells to treat a SLE patient who was resistant to various existing therapies [173]. This approach resulted in the complete elimination of circulating B cells and led to clinical and serological remission for the patients. An investigational trial is being conducted to evaluate the safety, tolerability, initial therapeutic (preliminary efficacy), pharmacokinetic (PK) profile, and pharmacodynamic (PD) responses of CD19-directed chimeric antigen receptor (CAR)-T cell therapy in patients with refractory or moderately to severely active SLE. The recruitment pool consists of these challenging patient populations who have not responded adequately to conventional treatments. Participants will undergo an administration of CD19-specific CAR-T cells, followed by a comprehensive monitoring period extending up to two years post-enrollment, thereby providing a thorough assessment of the therapy’s long-term effects. This rigorous examination aims to uncover potential benefits and risks associated with this innovative approach in managing SLE (NCT06106906).

CD22, a member of the sialoadhesin subclass within the Ig superfamily, functions as a lectin-like adhesion receptor and is integral to the B-cell activation complex [174]. This receptor is present on cells of the B lineage, spanning from immature B cells through to those in germinal centers, yet it is significantly lacking in plasma cells and memory B cells. When the BCR is stimulated, CD22’s trio of tyrosine-based inhibitory motifs (ITIMs) undergo phosphorylation. This process triggers the recruitment of tyrosine phosphatase I (SHP-1) and other regulatory factors, which in turn modulate and constrain the BCR signaling pathway [167, 174].Variations and polymorphisms in the CD22 gene have been correlated with an increased susceptibility to autoimmune conditions, such as SLE [175]. Research conducted on mice prone to autoimmunity has further established a connection between the CD22 gene and the development of SLE [176, 177]. Moreover, deficiencies in CD22 have been associated with elevated levels of autoantibody generation [177, 178].

Epratuzumab, a mAb targeting CD22, modulates B cell activity by triggering the phosphorylation of CD22. This initiates a cascade that results in the internalization of CD79α and CD22, followed by the downregulation of CD21, CD19, and CD79β from the cell surface through a process known as trogocytosis [179]. In the phase 2b EMBLEM trial, patients who received epratuzumab at various doses showed a higher rate of BICLA responses compared to those on placebo [180]. However, the phase 3 EMBODY 1 and 2 trials did not achieve their primary endpoints for epratuzumab [181]. Despite this, post hoc analyses of the EMBODY trials indicated beneficial effects in patients with SLE who also had Sjogren’s syndrome.

CD38, a type II glycoprotein, exhibits robust and consistent expression on plasma cells and plasmablasts that produce antibodies. In an ex vivo study assessing CD38 expression on various immune cells within peripheral blood mononuclear cells (PBMCs) from SLE patients, plasma cells and plasmablasts were noted to have the highest levels of CD38 expression, succeeded by natural killer (NK) cells, pDCs, a subset of regulatory T cells, and naive T cells [182]. These observations imply that CD38 represents an appropriate target for therapeutic exploration in SLE [183].

Daratumumab, a humanized mAb targeting CD38, was first approved for the treatment of multiple myeloma and is currently being investigated for SLE and other conditions. The mechanism of action involves binding to CD38 on target cells, leading to cytotoxic effects through multiple pathways including antibody-dependent phagocytosis (ADP), complement-dependent cytotoxicity (CDC), antibody-dependent cellular cytotoxicity (ADCC), induction of apoptosis, and modulation of immune responses. A case study involving the use of daratumumab, a monoclonal antibody targeting CD38, in SLE has shown early signs of therapeutic efficacy and validated its mechanism of action. In this instance, two individuals suffering from severe and life-threatening SLE exhibited notable clinical improvements, which were correlated with substantial reductions in autoantibody levels, decreases in plasmablast counts, and a dampening of type I IFN activity [184]. Additionally, the beneficial effects of daratumumab have been documented in other conditions driven by autoantibodies, including primary Sjögren’s disease, antineutrophil cytoplasmic antibody (ANCA)-associated vasculitis, and immune thrombocytopenia. This highlights the critical involvement of CD38 in the pathogenesis of autoimmune conditions [185].

Mezagitamab, known also as TAK-079, is an experimental human immunoglobulin G1 (IgG1) mAb with a high-affinity binding to CD38. Early non-clinical and initial human data from studies involving patients with multiple myeloma have shown mezagitamab to have a favorable safety profile and promising pharmacodynamic effects, particularly in the reduction of CD38-expressing target cells [186, 187]. These findings have bolstered the rationale for exploring mezagitamab as a potential treatment for SLE, a condition noted for the frequent presence of cells exhibiting aberrant CD38 expression. The phase 1b clinical trial, identified as NCT03724916, evaluated the safety profile, pharmacokinetic behavior, and pharmacodynamic effects of mezagitamab in participants suffering from moderate to severe SLE. This phase 1b clinical study established the favorable safety profile of mezagitamab, along with its anticipated pharmacodynamic effects and promising mechanistic outcomes in individuals with moderate to severe SLE. These results advocate for further exploration of mezagitamab as a potential therapeutic agent in the context of autoimmune diseases [188].

BAFF, also recognized as B-lymphocyte stimulator (BLyS), a 285-amino acid type-II transmembrane protein belonging to the tumor necrosis factor (TNF) ligand superfamily acts as a cytokine crucial for B cell proliferation and development. Elevated levels of BAFF are associated with an expansion of B cells, while the genetic elimination or pharmacological neutralization of BAFF results in a decrease in B cell numbers [189]. Binding to receptors such as BAFF-R, B Cell Maturation Antigen (BCMA), and Transmembrane Activator and Calcium-modulator and cyclophilin ligand (CAML) Interactor (TACI), BAFF exerts its effects [190]. BAFF-R is primarily expressed in immature B cells, whereas BCMA and TACI are more commonly associated with mature B cells and plasma cells. Another protein, A Proliferation-Inducing Ligand (APRIL), shares similarity with BAFF and functions parallelly in promoting B cell survival and development, while also binding to the BCMA and TACI receptors [190, 191]. The therapeutic importance of targeting BAFF-R is highlighted by the use of anti-BAFF-R therapies in managing myeloma, leukemia, and lymphoma, rendering BAFF-R an appealing therapeutic target in SLE as well [192].

Belimumab, a human monoclonal antibody that targets BAFF, marked a milestone as the first biological agent approved by the Food and Drug Administration (FDA) for the treatment of active SLE. Numerous clinical trials have consistently demonstrated its efficacy in reducing disease activity, mitigating the risk of severe flares, and decreasing reliance on corticosteroids [193–195]. Belimumab acts by binding and neutralizing soluble BAFF, thereby inhibiting its interaction with BCMA, TACI, and BR3 and consequently blocking the BAFF activity [196].The phase III trials, namely BLISS-North East Asia (NEA), BLISS-52, and BLISS-76, established that the intravenous administration of 10 mg/kg belimumab in conjunction with standard care was more effective in reducing disease activity than placebo [197, 198]. Post hoc analyses from the BLISS trials indicated that belimumab effectively achieved Lupus Low Disease Activity State (LLDAS) [199, 200]. Furthermore, the NEA trial revealed that belimumab-treated patients had a significantly reduced risk of severe flares compared to the placebo group, with respective rates of 22% and 12% (*p* = 0.0004) [193]. A closer examination of the data showed that the efficacy of the 1 mg/kg belimumab dosage was comparable to the 10 mg/kg dosage regarding both LLDAS and SLE Responder Index-4 (SRI-4), particularly in the BLISS-76 trial with extended follow-up period [199]. Also, a retrospective analysis highlighted that belimumab can be beneficial early in the disease for patients with active SLE and minimal baseline damage [201]. At present, a phase IV multicenter, randomized, double-blind, placebo-controlled clinical trial is underway, with the objective of investigating the efficacy of low-dose belimumab in preventing disease flares in SLE patients exhibiting low disease activity. This study seeks to expand our understanding of the therapeutic potential of belimumab, further delineating its role in the management of SLE in a subset of patients characterized by controlled disease states (NCT04515719).

Ianalumab, a monoclonal antibody, exhibits a dual mechanism of action by targeting BAFF-R and effectuating the deletion of peripheral BAFF-R + B cell [202]. Currently, there is an ongoing randomized, double-blind, placebo-controlled multicenter phase III clinical study evaluating the efficacy, safety, and tolerability of lanalumab in patients with lupus erythematosus, in addition to standard care treatment (NCT05624749). A phase 2 clinical trial (NCT03656562), encompassing a randomized, double-blinded, and placebo-controlled design, is presently underway to assess the safety and efficacy of lanalumab in 107 individuals ranging from 18 to 75 years old with SLE. The primary objective of this study is to evaluate the attainment of SRI-4 at week 53. Furthermore, an ongoing investigation in the form of a double-blinded, randomized, placebo-controlled, and multicenter three-arm study (NCT06133972) aims to evaluate the efficacy and safety of subcutaneous administration of lanalumab every 4 weeks or every 12 weeks, compared to placebo administered subcutaneously every 4 weeks. This study specifically targets adults with active LN. Upon completion, these trials are expected to yield substantial understanding of the role of anti-BAFF-R therapies in SLE patients. The SIRIUS-SLE 2 trial, a randomized, double-blind, placebo-controlled, multi-center, phase 3 study (NCT05624749), is devised to meticulously appraise the efficacy, safety, and tolerability of adjunctive lanalumab therapy in conjunction with standard care in individuals afflicted with SLE. This study endeavors to furnish comprehensive insights into the therapeutic value and safety profile of Ianalumab as an enhancement to the gold-standard SLE interventions.

Tabalumab, a human IgG4 mAb targeting BAFF, which does not only bind soluble BAFF but also membrane-bound BAFF, has been evaluated in two substantial phase III clinical trials conducted with double-blind, randomized controlled methodologies. In the initial trial, neither the primary nor the secondary endpoints were achieved, even though a notable decrease in anti-dsDNA antibody levels was observed among participants who received the treatment [203]. The second trial involved patients who were administered one of two doses of tabalumab or a placebo; it showed that the group with more frequent dosing met its primary outcome [204]. However, the efficacy in terms of response rate did not surpass the results noted in trials featuring belimumab. Additionally, critical secondary endpoints, such as the time to severe flare, the potential for corticosteroid reduction, and the impact on fatigue, were not achieved. Given these trials’ outcomes, which did not demonstrate a compelling advantage for tabalumab, the sponsor decided to discontinue its further development for the treatment of SLE.

Atacicept is a fusion protein that combines the TACI receptor, a BAFF receptor, with the Fc region of IgG. This construction can bind and neutralize both BAFF and APRIL, suggesting it might offer enhanced potency and efficacy compared to treatments that target BAFF alone. In the APRIL-SLE trial, participants were randomized to receive atacicept at doses of 75 mg or 150 mg, or a placebo, twice weekly for 48 weeks [205]. The 150 mg dosage group was discontinued early due to two reported deaths, and the 75 mg dose did not differ significantly from placebo in the primary outcomes of flare rate or time to the first flare. However, a significant reduction in both flare rate and time to the first flare was noted among patients who received the higher 150 mg dose of atacicept. In the phase IIb ADDRESS II study, individuals with active SLE were assigned to receive 75 mg or 150 mg of atacicept, or a placebo [206]. Although the study’s main goal of achieving an SRI-4 response was not reached, there was a noticeable trend toward a higher response rate with atacicept, particularly among patients exhibiting high disease and serological activity. The drug exhibited a favorable safety profile in both the ADDRESS II trial and its subsequent extension. In contrast to the APRIL-SLE trial, atacicept did not show an increased frequency of adverse events or serious infections when compared to the placebo [206]. Consideration is being given to phase III trials for SLE involving atacicept.

Telitacicept, also known as RC18, is a BAFF antagonist that is also a recombinant fusion protein comprising the Fc domain of human IgG1 and the extracellular domain of the TACI receptor. This molecule has demonstrated efficacy in a phase 2b trial, achieving its primary endpoint with an SRI-4 response across all tested doses. Currently, a phase 3 trial of telitacicept has been concluded (NCT04082416).

##### Targeting T cells as a therapeutic strategy for SLE

The therapeutic strategies aimed at the T cell compartment include co-stimulation blockade, cytokine blockade and kinase inhibition (Table 3; Fig. 3).

**Table 3:** T-cell targeted therapies in SLE

Type of agent	Target	Agent	Stage of development	Reference
Co-stimulation blockade	CD40–CD40L	BI655064	phase II trial	NCT02770170
	CD40–CD40L	VAY736	phase II trial	NCT03656562
	CD40–CD40L	Dapirolizumab pegol	phase IIb trail phase III trial	[207] NCT04976322
	CD40–CD40L	CDP7657	phase I trials	NCT01093911 NCT01764594
	CD80/86–CD28	CTLA4-Ig	Phase I/II trail	NCT02429934
	ICOS–ICOSL	AMG 557	phase I trials	NCT01683695 NCT00774943 NCT02391259
Cytokine blockade	IL‑6R	Tocilizumab	phase I trial	NCT00046774
	IL‑6R	ALX-0061	phase II trial	NCT02437890
	IL‑6	CNTO 136	phase I trial phase II trial	NCT01702740 NCT01273389
	IL‑12 and IL‑23	Ustekinumab	phase III trials	NCT03517722 NCT04060888
	IL‑23	Guselkumab	phase II trial	NCT04376827
	IL-17	Secukinumab	phase II trial	NCT03866317
	IFN-γ	AMG 811	phase I trials	NCT00818948 NCT02291588
Kinase inhibition	JAK	Filgotinib	phase II trial	NCT03285711
	JAK	Upadacitinib	phase II trial	NCT04451772
	JAK	Deucravacitinib	phase II trial phase III trials	NCT03920267 NCT05617677 NCT05620407
	JAK	Tofacitinib	phase Ib trail	NCT02535689
	JAK	Baricitinib	phase II trial	NCT02708095
	JAK	lanraplenib	phase II trials	NCT03134222 NCT03285711
	JAK	Brepocitinib	phase II trial	NCT03845517
	JAK	solcitinib	phase II trial	NCT01777256
	JAK	baracetinib	phase III trial	NCT05432531
	Calcineurin	Voclosporin	phase II trials phase III trials	NCT05306873 NCT02949973 NCT02141672 NCT03597464 NCT06406205 NCT03021499 NCT05288855 NCT05962788
	mTOR	Sirolimus	phase II trial	NCT00779194
	mTOR	INK128	phase II trial	NCT00775476

Approximately a dozen experimental drugs are being developed to target co-stimulatory molecules. Dapirolizumab pegol [207], iscalimab, and ruplizumab are examples of agents that target the CD40L-CD40 signaling axis, while dazodalibep represents a next-generation fusion protein intended to inhibit CD40 ligand (CD40L). Additionally, the CD28 molecule, a T cell costimulatory factor crucial for the activation of pathogenic T cells in autoimmune conditions, is being targeted by three investigational drugs: theralizumab, lulizumab pegol, and acazicolcept, which also targets the ICOS pathway. Lastly, LY3361237 is a novel B- and T-lymphocyte attenuator (BTLA/CD272) agonist, representing a first-in-class approach in this area.

Given that patients with SLE exhibit increased expression of CD40, the blockade of CD40 receptor presents a potential avenue for modulating the immunologic and clinical activity of SLE.

BI655064 is classified as a humanized anti-CD40 mAb. In a phase 2 trial with a randomized, double-blind, and placebo-controlled design, the safety and efficacy of BI655064 were assessed in patients with active LN across three dose levels: 240 mg, 180 mg, and 120 mg (NCT02770170). A total of 121 participants aged between 18 and 70 were allocated to receive either subcutaneous injections of placebo or BI655064. The primary outcome, complete renal response (CRR) at week 52, was achieved by 48.3% in the placebo group, 38.3% in the 120 mg group, 45.0% in the 180 mg group, and 44.6% in the 240 mg group. Because of a notable placebo response observed in the study, a post-hoc analysis was undertaken to verify the endpoint’s validity, which indicated a 15% effect size in the 180 mg dosage group. There were serious adverse events (SAEs) in 22 of the 81 patients who received BI 655,064, compared to 8 out of 20 patients in the placebo group. Currently, the future development plans for this compound are not publicly determined [208, 209].

Iscalimab, a fully human anti-CD40 mAb, has been investigated in various medical conditions, including Sjogren’s syndrome, cancer, and kidney transplant patients. At present, a meticulously designed study (NCT03656562) is underway to comprehensively evaluate the dynamic interplay between pharmacokinetics and pharmacodynamics, alongside the safety profile, tolerability, and tentative therapeutic impact of VAY736 (ianalumab) and CFZ533 (iscalimab) in participants diagnosed with SLE. This investigation adopts a placebo-controlled, double-blind, randomized parallel-group methodology to ensure rigorous assessment while mitigating bias. Throughout this inquiry, a primary objective is to ascertain a nuanced understanding of the investigation agents’ action mechanism, tolerability, and potential efficacy in alleviating the manifestations of SLE - all of which are integral to paving the pathway for innovative therapeutic avenues in the management of this complex autoimmune condition.

Dapirolizumab pegol (DZP) represents an emerging therapeutic option for SLE, formulated as a PEGylated anti-CD40 ligand Fab’ antibody fragment. This agent functions by binding to CD40, a key molecule in the activation of immune cells, thereby dampening inflammation and alleviating SLE symptoms [210]. In a phase IIb clinical trial, DZP demonstrated efficacy in reducing disease activity indices, lowering anti-double-stranded DNA (anti-dsDNA) antibody levels, and restoring C3 and C4 complement levels versus placebo at the 24-week mark [207]. Nevertheless, the highest dosage of DZP, specifically 45 mg/kg, was associated with a modestly increased rate of severe treatment-emergent adverse events (TEAEs) compared to other dosage groups [207]. Given the potential for thromboembolic risks associated with CD40L-targeting therapies [211], it is significant that DZP has shown a generally favorable safety profile, exhibiting good tolerability and a comparatively lower risk of thromboembolic events compared to other mAbs targeting CD40L [212]. DZP is currently under investigation in phase III clinical trials for SLE (NCT04976322).

A phase I clinical trial (NCT01093911) is completed, evaluating CDP7657, which is a monovalent, PEGylated Fab fragment of an anti-CD40L antibody, for its use in SLE. Given that individual IgG molecules are incapable of binding to or activating the platelet FcγRIIa receptor—this receptor is only triggered by clustered, multimeric IgG immune complexes [54]—this specific antibody format may offer a reduced risk of thromboembolic events, thus potentially providing a safer therapeutic option. A multicenter, randomized, double-blind, placebo-controlled, parallel-group, and repeat-dose study (NCT01764594) was also initiated to evaluate the impact of CDP7657 in individuals with active SLE. This trial was designed to assess the efficacy and safety of the intervention under rigorous scientific scrutiny.

The traditional CD80/CD86-CD28 costimulatory pathway represents a key target for immunotherapeutic interventions in autoimmune diseases. Cytotoxic T-lymphocyte-associated protein 4 (CTLA-4), a negative regulator that is upregulated on activated T cells to prevent excessive activation, binds more avidly to CD80/CD86 than CD28. This characteristic has made it a therapeutic contender for dampening T-cell responses by competitively inhibiting CD28. CTLA4-Ig, a fusion protein, has been extensively tested as an immunotherapy across various disease systems and models [213, 214]. In the context of SLE, CTLA4-Ig has emerged as a promising therapeutic option, with promising preclinical findings from multiple models [215]. However, its clinical efficacy is still under investigation, as results from a few phase II/III clinical trials are pending.

Co-stimulation of lymphocytes is a pivotal aspect of immunology, critical for regulating inflammation and guiding immunotherapeutic strategies [216–219]. The inducible T cell co-stimulator (ICOS) on T cells increases after interaction with peptide-major histocompatibility complex (pMHC) in conjunction with CD28 co-stimulation. This engagement leads to the binding of ICOS with its unique ligand, the inducible T-cell co-stimulatory ligand (ICOSL), alternatively termed B7-related protein-1 or B7h. This binding initiates essential T cell functions, such as cytokine secretion and the specification of T cells into the Tfh lineage, which is crucial for B cell help and antibody production, over other effector lineages [220, 221].

AMG 557 is a humanized IgG2 mAb that specifically targets the ICOSL. By binding to ICOSL, it inhibits the functional engagement with its receptor ICOS on activated T cells. Importantly, AMG 557 exhibits no cross-reactivity with other members of the B7 family of co-stimulatory molecules.

Tocilizumab is a humanized mAb that targets the α-chain of the IL-6 receptor, thereby blocking the interaction of IL-6 with both membrane-bound and soluble forms of the IL-6 receptor. Its safety and efficacy have been assessed in clinical trials for conditions such as rheumatoid arthritis, juvenile idiopathic arthritis, and Castleman’s disease [222]. In a Phase I, open-label, dose-escalation study (NCT00046774), 16 individuals with mild to moderate SLE disease activity received tocilizumab bi-weekly for 12 weeks at one of three dosages (2 mg/kg for 4 participants, 4 mg/kg for 6 participants, and 8 mg/kg for 6 participants). These patients were then monitored for an additional 8 weeks. This preliminary investigation offers initial evidence that tocilizumab can successfully inhibit IL-6 in SLE patients. The observed enhancements in inflammatory biomarkers and the clinical and serological indicators of lupus activity are promising. Further exploration in controlled trials is necessary to substantiate these findings and to evaluate the potential of tocilizumab in SLE treatment [223].

A Phase II multicenter clinical trial (NCT02437890) is designed to evaluate the safety and efficacy of vobarilizumab (ALX-0061) when administered subcutaneously in individuals with moderate to severe active SLE. The primary objective of this randomized, double-blind, placebo-controlled, dose-ranging study is to compare the efficacy and safety of various subcutaneous dosages of ALX-0061 to placebo in subjects with moderate to severe active, seropositive SLE. Secondary objectives include assessing the pharmacokinetics (PK), pharmacodynamics (PD), immunogenicity, flare rate, potential for steroid reduction, and the impact on health-related quality of life associated with different dosage regimens of ALX-0061. This comprehensive evaluation aims to determine the optimal dosing strategy for ALX-0061 in the treatment of SLE.

Sirukumab, previously known as CNTO136, is a human monoclonal antibody that targets IL-6 with high affinity and specificity. It neutralizes IL-6 by inhibiting STAT-3 phosphorylation, thereby reducing IL-6’s biological effects. Initial human studies in healthy volunteers indicated that a single intravenous dose of sirukumab, ranging from 0.3 to 10 mg/kg, was generally well tolerated without any dose-related safety concerns [224]. A two-part, Phase I clinical trial (NCT01702740), characterized by randomization, double-blinding, and placebo control, assessed the safety and pharmacokinetics of multiple intravenous doses of sirukumab across three escalating dosage levels in 31 patients with cutaneous lupus erythematosus (CLE) and at a single dosage level in 15 patients with SLE. Overall, the participants in this study presented with mild, stable, yet active disease [225]. Sirukumab therapy was generally well tolerated among patients with both CLE and SLE. Patients who received sirukumab exhibited sustained, stable reductions in white blood cell count, absolute neutrophil count, and platelet count, which were not dose dependent. These reductions are consistent with the known mechanism of action of sirukumab and the role of IL-6 in hematopoiesis [226]. A similar effect has been observed with tocilizumab, another anti-IL-6 receptor antibody [223].

A multicenter, randomized, double-blind, placebo-controlled, parallel-group study (NCT01273389) is completed to evaluate the efficacy and safety of Sirukumab in patients with active LN. The trial is divided into three phases: a screening period of approximately 8 weeks leading up to randomization, a 24-week treatment phase, and a follow-up phase extending to week 40. During an 8-week run-in period, the stability of baseline renal parameters will be confirmed before the administration of the first study medication. The objective of this trial is to assess the intravenous administration of Sirukumab in patients with active International Society of Nephrology/Renal Pathology Society Class III and IV LN. This proof-of-concept trial failed to establish the expected therapeutic benefits of sirukumab and did not meet the required safety standards for patients with active lupus nephritis who were concurrently undergoing immunosuppressive therapy [227].

Memory T cells are categorized into several subsets, with Th1 cells being crucial for the initiation of cell-mediated immunity, and Tfh cells being vital for the differentiation and activation of B cells. The cytokine IL-12 is instrumental in the development of Th1 and Tfh cells in humans, and increased levels of serum IL-12 are observed in individuals with active SLE [228]. Ustekinumab, a monoclonal antibody targeting IL-12/IL-23 (p40), has been approved for treating psoriasis and psoriatic arthritis, with its efficacy and safety having been assessed. In a phase IIb global clinical trial that included patients with highly active SLE, Ustekinumab (UST) was compared to a placebo. The trial’s primary endpoint was the SRI-4 response rate at 24 weeks, which was significantly higher in the ustekinumab group compared to the placebo group. This beneficial effect was sustained for up to one year, with no significant adverse events reported [229].

A Phase 3, multicenter, randomized, placebo-controlled trial (NCT03517722) is to evaluate the efficacy and safety of ustekinumab in individuals with SLE. Eligible participants with active SLE, defined by a SLEDAI 2000 score of at least 6 during the screening phase and a SLEDAI-2 K score of at least 4 at week 0, were enrolled despite ongoing treatment with oral glucocorticoids, antimalarial agents, or immunomodulatory therapies. They were then randomly assigned in a 3:2 ratio to receive either ustekinumab (intravenous infusion at approximately 6 mg/kg at week 0, followed by subcutaneous injections of 90 mg every 8 weeks starting at week 8) or a placebo, continuing through week 48. The primary outcome measure is the SLE Responder Index (SRI)-4 at week 52, with key secondary outcomes including the time to flare and the achievement of SRI-4 at week 24. Regrettably, the trial results indicated that ustekinumab did not outperform placebo in the cohort of adults with active SLE. The adverse events observed were in line with the established safety profile of ustekinumab, leading to the premature termination of the study [230].

Another phase 3, multicenter, randomized, double-blind, placebo-controlled, parallel-group study (NCT04060888) of Chinese patients with SLE is evaluating the efficacy and safety of Ustekinumab. The objective of this study was to assess the efficacy of ulinastumab in Chinese patients with SLE, who do not respond well to one or more standard therapies. The study design included the randomization of participants to groups that received either ulinastumab (approximately 6 mg/kg intravenously at week 0, followed by 90 mg subcutaneously at week 8 and every 8 weeks thereafter) or placebo; The study will continue until week 48. The primary end point was the SLE response index (Sri-4) at week 52, and the primary secondary end points included the time to exacerbate at week 52 and Sri-4 at week 24.

Guselkumab is a mAb that exhibits high-affinity binding to human IL-23, preventing the interaction of extracellular IL-23 with its cell surface receptor. This blockade impedes the intracellular signaling pathways specific to IL-23, thereby inhibiting the activation and cytokine production associated with IL-23. LN, a renal manifestation of SLE, represents a significant area in need of novel and effective treatment strategies that can offer superior long-term outcomes compared to existing options. A Phase 2 study (NCT04376827) aims to assess the safety and efficacy of guselkumab when administered in conjunction with the standard-of-care, in comparison to placebo plus standard-of-care. The study’s total duration spans up to 68 weeks, which includes a screening phase of up to 8 weeks, a 48-week double-blind treatment phase, and a 12-week post-treatment safety follow-up phase. Participants who reach the 52-week mark with a complete renal response (CRR) may be eligible to enroll in a long-term extension (LTE) of the study, extending their involvement through to Week 152, followed by a final 12-week safety follow-up visit.

IFN-α is recognized as a key cytokine in the pathogenesis of SLE. A multitude of studies have demonstrated that IFN-α stimulates the production of interleukin-17 (IL-17), which has been identified as a significantly proinflammatory cytokine in SLE [231, 232]. Discoid lupus erythematosus (DLE) represents a dermatological presentation of lupus, which may occur as an aspect of SLE or as a standalone chronic skin condition without systemic manifestations. Secukinumab, marketed as Cosentyx, is a monoclonal antibody directed against interleukin-17 A (IL-17 A). Given its established safety, its significant efficacy in treating psoriasis and in conditions unresponsive to steroid therapy, and the immunohistochemical data suggesting a role for IL-17 A in the inflammatory process of DLE, researchers are proposing a pilot study(NCT03866317) to investigate secukinumab’s potential in managing discoid lupus erythematosus.

Interferon-gamma (IFN-γ) is a multifunctional type II interferon predominantly generated by effector CD4 + T cells of the Th1 lineage, cytotoxic CD8 + T cells, and NK cells. It is also produced, albeit to a lesser extent, by other immune cells including DCs, macrophages, and B lymphocytes. Research has demonstrated that SLE patients exhibit elevated serum levels of IFN-γ compared to healthy controls [233–236]. Moreover, an abnormal accumulation of IFN-γ occurs within the body well in advance of an SLE diagnosis, preceding the emergence of autoantibodies and interferon-alpha (IFN-α) [237].

Clinical trials focused on targeting IFN-γ have begun to show promise. AMG 811, a fully human mAb of the IgG1 subtype against IFN-γ, has demonstrated good tolerability in patients with mild to moderate SLE. A single administration of AMG 811 has been shown to normalize the expression of IFN-regulated genes, leading to a dose-dependent reduction in serum CXCL-10 levels [238, 239]. While AMG 811 modulated IFN-γ-associated biomarkers and demonstrated a favorable safety profile, it did not yield significant clinical improvements in patients with DLE [240]. Nonetheless, positive outcomes from phase Ib trials have highlighted the potential of blocking the IFN-γ pathway in treating extrarenal manifestations of lupus [241]. These collective findings position IFN-γ as a pivotal cytokine in LN, and further investigation into IFN-γ inhibition in LN is warranted, considering the tolerable safety profile associated with its targeted blockade. Two concluded studies, identified by the trial numbers NCT00818948 and NCT02291588, have assessed the safety profile of AMG118 in the context of SLE treatment.

The JAK/STAT signaling pathway is a crucial cellular mechanism that responds to a broad spectrum of cytokines and growth factors [242]. Numerous inflammatory cytokines, which are involved in the pathogenesis of SLE, such as type I and II IFNs, utilize the JAK-STAT pathway for signaling [243, 244]. Inhibitors of Janus kinase (JAK inhibitors, or jakinibs) have shown promise in various lupus models in mice [245]. Clinical trials utilizing jakinibs have demonstrated efficacy in treating arthritis in individuals with mild-to-moderate SLE [246, 247].

In the context of treating SLE with JAK inhibitors, global phase II or III trials are presently being conducted on filgotinib (NCT03285711), Upadacitinib (NCT04451772), and deucravacitinib (BMS-986165). Notably, deucravacitinib, a TYK2 inhibitor, has drawn significant interest. Unlike other JAK inhibitors that competitively bind to adenosine triphosphate (ATP) sites, deucravacitinib is an allosteric inhibitor, thus is anticipated to exhibit high specificity. In a phase II trial involving patients with active SLE (NCT03920267), the primary endpoint a SRI-4 response rate at week 32 was markedly higher in individuals administered deucravacitinib at 3 mg twice daily (58%) compared to those given a placebo (34%). Additionally, numerous secondary endpoints were achieved [248]. Currently, phase III trials are underway (NCT05617677, NCT05620407). Deucravacitinib has also been shown to modulate type I IFN-associated gene expression. Considering that one possible reason for clinical trial failures is the inhibition of regulatory T cell differentiation and activation due to the suppression of IL-2 signaling pathways by baricitinib, deucravacitinib, which preserves IL-2 signals, may offer promising efficacy.

Tofacitinib, a medication under investigation, has demonstrated the ability to modulate irregular neutrophil behavior and type I IFN reactions in a mouse model of lupus, as well as endothelial dysfunction and lipoprotein levels [245]. Furthermore, tofacitinib was evaluated in a Phase Ib clinical trial (NCT02535689) that enrolled patients with mild to moderate SLE, stratified based on their STAT4 genetic risk profile. The trial’s primary focus was on assessing the tolerability and safety of tofacitinib, with secondary endpoints evaluating clinical responses and conducting mechanistic analyses. The study demonstrates that tofacitinib is both well-tolerated and safe in mild-to-moderate SLE individuals. It reports no unexpected adverse events, no exacerbation of SLE disease activity, and no occurrences of severe adverse events, thromboembolic events, or opportunistic infections associated with tofacitinib treatment. Furthermore, treatment with tofacitinib has been shown to enhance high-density lipoprotein (HDL) cholesterol levels and particle numbers, lecithin: cholesterol acyltransferase levels, and cholesterol efflux capacity. It also ameliorates endothelium-dependent vasorelaxation and arterial stiffness. Tofacitinib significantly reduced the presence of circulating NETs, the levels of low-density granulocytes, and the type I IFN gene signature. These improvements were particularly pronounced in SLE patients carrying the STAT4 risk allele. Further long-term studies are required to ascertain the efficacy of tofacitinib in preventing cardiovascular disease (CVD) in SLE patients [249]. Baricitinib, a selective and reversible inhibitor [250] of JAK1 and JAK2, is an orally administered drug that has been approved for treating moderate-to-severe active rheumatoid arthritis in adults across more than 75 countries, including the United States, Japan, and several European Union nations. By inhibiting JAK1/JAK2, baricitinib potentially modulates the production of proinflammatory cytokines, including type I IFNs, IFN-γ, IL-6, IL-12, and IL-23 [247, 251]. In a Phase II clinical trial (NCT02708095) investigating the effects of baricitinib in individuals with SLE, the administration of 4 mg of daily oral baricitinib in conjunction with standard care demonstrated superior disease activity improvement at 24 weeks compared to placebo with standard care [247, 252]. However, the study did not record any significant overall least squares (LS) which mean changes from baseline in conventional serologic biomarker levels, such as anti-dsDNA antibodies, complement component 3 (C3), or complement component 4 (C4), with baricitinib treatment. The conclusions derived from this study are subject to certain limitations. The findings do indicate a potential benefit of baricitinib for SLE treatment, but the study’s 24-week duration is insufficient to evaluate its effectiveness over a more extended period. Furthermore, the limited number of participants in this Phase II trial may affect the robustness of the analysis. Additional data from three ongoing Phase III trials (NCT03616912, NCT03843125, and NCT03616964) are expected to provide a more detailed understanding of the drug’s long-term benefits and safety profile.

Meanwhile, another two studies treated moderate to severe cutaneous lupus erythematosus (NCT03134222) as well as those afflicted with membranous lupus nephropathy (NCT03285711) with filgotinib or lanraplenib, to investigate the safety and efficacy of the Janus kinase 1 inhibitor filgotinib (FIL) and the spleen tyrosine kinase inhibitor lanraplenib (LANRA) in the treatment of cutaneous lupus erythematosus (CLE). They are phase 2, randomized, double-blind, placebo-controlled, exploratory, proof-of-concept investigations, evaluating the efficacy of LANRA (30 mg), FIL (200 mg), or placebo (PBO) administered once daily over a 12-week period in patients diagnosed with active CLE. Following the initial 12-week interval, participants originally assigned to the placebo group were re-randomized at a 1:1 ratio to commence treatment with either LANRA or FIL for an additional period extending up to 36 weeks [253].

The mammalian target of rapamycin (mTOR) pathway is widely acknowledged for its role in regulating cellular survival, metabolism, and proliferation [254]. The activation of this pathway has been implicated in the development of SLE [255, 256], with both the activation of mTOR and the potential for therapeutic intervention being observed in T cells within the context of SLE [257–261]. Rapamycin, known for its efficacy in suppressing antigen-induced T-cell proliferation [262], has been formulated as sirolimus to prevent organ transplant rejection. Sirolimus binds with high affinity to its cellular receptor, FKBP12, a 12-kilodalton protein that is notably overexpressed in T cells of individuals with lupus [263]. The sirolimus-FKBP12 complex inhibits the activation of the mammalian target of mTOR [264]. In vivo studies have shown that sirolimus can effectively eliminate autoimmunity in mice prone to lupus [265, 266]and has been shown to suppress disease activity in a retrospective analysis of patients with SLE [267].

A study (NCT00779194) aims to evaluate the tolerability, safety, and the metabolic, immunological, and therapeutic impacts of sirolimus in patients with severe SLE who are either intolerant of or unresponsive to conventional treatment options. This mechanistic study offers initial indications that sirolimus is safe, well tolerated, and demonstrates clinical efficacy in SLE patients under vigilant monitoring for reversible oral ulcers, headaches, and cytopenia. The disease activity, as measured by the reversal of pro-inflammatory T-cell lineage specification, exhibited a positive trend at visit 6, corresponding to 12 months of sirolimus therapy [130].

The mTOR inhibitor INK128 is an orally administered, potent, and selective ATP competitor that targets both mTORC1 and mTORC2 complexes. However, there are no clinical studies on INK128 for SLE. A phase II study (NCT00775476) using N-acetylcysteine to treat SLE is being recruited.

Voclosporin, a novel calcineurin inhibitor (CNI), has received approval for the treatment of adult lupus nephritis patients [268] and has demonstrated enhanced complete renal response rates in a phase 2 trial. This phase 2b international study was a randomized, blinded, placebo-controlled trial that evaluated the effects of two different doses of voclosporin, in conjunction with mycophenolate mofetil and steroids, against a placebo. The group receiving 23.7 mg of voclosporin twice daily exhibited a notably higher 6-month complete renal response rate compared to the placebo group. This dosage of voclosporin was subsequently investigated in a phase 3 clinical trial. The phase 3 trial was a randomized, placebo-controlled study designed to assess the efficacy and safety of voclosporin in lupus nephritis patients. The study achieved all primary and secondary endpoints with the use of voclosporin combined with mycophenolate mofetil and a rapidly tapered low-dose steroid regimen. The primary endpoint was the complete renal response at 52 weeks, marking a longer duration than prior trials of CNIs for lupus nephritis. The voclosporin group’s improved efficacy was realized with a steroid regimen that led to a significantly lower cumulative steroid exposure than in any prior study. The outcomes of this phase 3 trial corroborate the efficacy demonstrated in the phase 2 trial and indicate an enhanced safety for voclosporin.

This study included patients who had a recent biopsy indicating active lupus nephritis or a biopsy from 6 months to 2 years ago that demonstrated lupus nephritis with current clinical signs of a renal flare, reflecting clinical practice in real-world settings. The findings indicated that voclosporin, when used in combination with MMF and low-dose steroids, demonstrated greater efficacy than placebo, reinforcing the early treatment response and overall effectiveness observed with voclosporin in the phase 2 trial [269]. Collectively, the outcomes from both the phase 2 and phase 3 clinical studies of voclosporin signify a significant step forward in the management of patients with active lupus nephritis.

#### Precision medicine

Given the pronounced heterogeneity of SLE, it has proven challenging to achieve uniform therapeutic outcomes across all patients through B cell-targeted therapies aimed at B cell depletion. However, it is notable that significant variability in treatment response was identified based on the expression levels of IFN signature genes, as observed in the global Phase IIb trial of anifrolumab [270]. This trial may represent an initial step towards precision medicine for SLE, which aims to stratify or subgroup patients to enhance diagnostic accuracy and treatment efficacy [271]. Precision medicine in SLE entails a tailored approach encompassing diagnosis, therapy, and disease management. As depicted in the 2019 EULAR/ACR classification criteria, SLE presents a spectrum of clinical features, serologic profiles, and immune system mechanisms [272]. These standardized criteria are instrumental in recognizing patient groups with common traits, thus enabling more focused research endeavors and individualized therapeutic strategies.

Since the completion of the Human Genome Project in 2003, genetic information has been instrumental in cancer treatment for both therapy and prognosis monitoring. Although our comprehension of SLE’s genetic pathology has advanced, the disease’s heterogeneity remains incompletely understood. It is anticipated that future therapeutic approaches will be tailored to the disease’s subgroups at the cellular and molecular levels, informed by data gleaned from “omics” analyses, including immunophenotyping [273].Omics technologies, such as genomics, transcriptomics, and proteomics, play a pivotal role in distinguishing between SLE patients who are likely to respond to treatment and those who may not, by pinpointing genetic and molecular differences. By leveraging these technologies, precision treatment in SLE can be tailored to an individual’s specific diagnosis and genetic makeup, leading to enhanced treatment outcomes and an improved quality of life, all while aiming to reduce the incidence of adverse effects.

## Future directions

### Challenges in SLE research and treatment

The pathogenesis of SLE is complex and involves immune system disorders, breakdown of immune tolerance, genetic predisposition to SLE and a range of environmental triggers. These complex triggers have brought great challenges to the study of the pathogenesis of SLE. The immune system’s self-reactive actions culminate in a range of clinical manifestations characteristic of SLE, including, but not limited to, skin eruptions, oral ulcerations, inflammatory arthritis, serositis, neuropsychiatric manifestations, glomerulonephritis, and hematological disorders [120]. The multifaceted nature of SLE and its tendency to involve multiple organs pose significant challenges to treatment, requiring a comprehensive understanding of the underlying immune pathogenesis to develop targeted and effective treatment strategies.

For many years, SLE has been treated mainly with non-specific approaches, including cytotoxic and immunosuppressive drugs, antimalarials, glucocorticoids and NSAIDs. While these treatment strategies are effective in mitigating the effects of the disease, they are not a complete cure and are often accompanied by adverse reactions. In recent years, however, the field of SLE treatment has evolved to include more precise strategies such as cellular therapy. These targeted therapeutic drugs aim to control and treat SLE by targeting B-cells and T-cells, inhibiting the activation and function of these cells, as well as the abnormal activation of the immune system. While some of these targeted drugs have achieved some clinical effects, many others have failed after multiple rounds of clinical trials. These phenomena raise the question of whether there exists a patient subgroup that requires a combined use of B-cell targeted and T-cell targeted drugs?

### Promising areas for future investigation

The complexity of SLE’s pathogenesis and clinical symptoms poses numerous challenges and difficulties in its treatment, necessitating deeper research and understanding of SLE’s pathogenesis. The combination of targeted therapy and precision medicine may be an important direction for future SLE treatment. By precisely identifying genetic and molecular differences and combining the latest cellular targeted therapeutic methods, it plays a key role in distinguishing SLE patients who may respond to treatment from those who may not. By leveraging these technologies, precision medicine for SLE can be tailored to an individual’s specific diagnosis and genetic makeup, thereby enhancing treatment efficacy and improving quality of life, while aiming to reduce the incidence of adverse reactions.

## Data Availability

Not applicable.
